# Ultrasound imaging findings in primary biliary cholangitis

**DOI:** 10.1186/s12876-023-03083-w

**Published:** 2023-12-19

**Authors:** Yuan Zhang, Xing Hu, Jing Chang, Weihua Li, Chunyang Huang, Haiping Zhang, Jianjun Shen, Ning Shang, Fankun Meng

**Affiliations:** 1 Department of Ultrasound, Youan Hospital, Capital Medical University, No 8, Xitoutiao, Youanmenwai, Fengtai District, Beijing, 100069 China; 2 Pathology Department, Youan Hospital, Capital Medical University, Beijing, China; 3 Institute of Hepatology, Youan Hospital, Capital Medical University, Beijing, China; 4 Second Department of Liver Disease Center, Youan Hospital, Capital Medical University, Beijing, China; 5 Clinical Laboratory Center and Clinical Research Center for Autoimmune Liver, Youan Hospital, Capital Medical University, Beijing, China; 6 Function Diagnosis Department, Handan Infectious Disease Hospital, Handan, China

**Keywords:** Ultrasonography, Primary biliary cholangitis, Hepatitis B, Hepatomegaly, Splenomegaly

## Abstract

**Background:**

Our study aimed to analyze the characteristics of ultrasound images corresponding to each histological stage of primary biliary cholangitis (PBC).

**Methods:**

We prospectively analyzed 75 confirmed cases of PBC and used liver biopsy as the gold standard to determine the disease stage.

**Results:**

The typical ultrasound images of patients with PBC were characterized by a thickening of the portal vein wall (PVW) and periportal hypoechoic band (PHB) width with increasing histological stages, and significant increases in the left hepatic lobe diameter (LHLD) in stage II (by 64.0%) and stage III (by 69.2%). PHB width (r = 0.857, *p* < 0.001), PVW thickness (r = 0.488, *p* < 0.001), and spleen area (r = 0.8774, *p* < 0.001) were positively correlated with the histological stage. Significant changes were noted in the liver surface, echo texture, and edge between different stages. The areas under the receiver operating characteristic curve of composite indicators were 0.965 for predicting progressive PBC(≥ stage 2), and 0.926 for predicting advanced PBC(≥ stage 3).

**Conclusions:**

The ultrasound imaging characteristics of patients with PBC varied according to the histological staging. LHLD, PVW thickness, and PHB width were significantly correlated with the histological stage. A combination of high- and low-frequency ultrasound imaging can provide relevant cues regarding the degree of PBC progression and important clinical reference values. The application of all the ultrasound image findings as the composite indicators can better predict progressive and advanced PBC, providing important clinical reference values.

**Supplementary Information:**

The online version contains supplementary material available at 10.1186/s12876-023-03083-w.

## Background

Primary biliary cholangitis (PBC) is a chronic idiopathic disorder, characterized by bile accumulation and progressive necrosis of the intrahepatic bile ducts. Unlike primary sclerosing cholangitis (PSC), which is characterized by fibrotic changes and narrowing of both intra- and extra-hepatic bile ducts, or autoimmune hepatitis (AIH), which primarily involves injury to the liver parenchymal cells without apparent damage to the bile ducts, PBC is confined to the small intrahepatic bile ducts. As the disease progresses, the affected bile ducts undergo a gradual degradation that may lead to their complete disappearance, resulting in fibrosis of the hilum and, eventually, cirrhosis. The average duration from symptom onset to death in patients with PBC is approximately 15–20 years [1], and therefore, evaluating the disease progression is of paramount importance for effective management and prognosis. Although liver biopsy is considered the definitive tool for PBC diagnosis and staging, it is used sparingly in clinical practice, with only 20% of patients with PBC undergoing liver biopsy [2]. Some studies have suggested that certain ultrasound imaging findings could be used as markers for the progression of liver fibrosis, including liver surface, parenchymal echoes, liver edge, and spleen size [3–5]. However, their suitability in the specific case of PBC has not been appropriately explored.

Several previous reports exist regarding specific magnetic resonance imaging (MRI) characteristics associated with PBC, which include the presence of a periportal halo sign, splenomegaly, and lymphadenopathy [6]. However, the literature on the potential use of ultrasound for diagnosing and monitoring PBC progression is scarce. There have been reports of low reflectivity around the portal vein in cases of PBC diagnosed by ultrasonography [7], and in the later stages of the disease, larger volumes of peri-hepatic lymph nodes have also been reported [8]. In addition, one of the most common chronic liver diseases in China (chronic hepatitis B [CHB]) is also associated with similar ultrasound imaging characteristics. Therefore, this study was aimed prospectively analyze and compare the differences in abdominal ultrasound imaging findings between PBC and CHB to determine specific features for each stage of PBC and thus allow for an efficient evaluation of the extent of disease progression in patients with PBC through ultrasonography.

## Methods

### Clinical data

This prospective study was approved by the Institutional Review Board, and informed consent was obtained from all participants. Seventy-five cases of PBC and 57 cases of CHB (104 women and 28 men) diagnosed by liver histopathology or clinically confirmed between February 2019 and August 2022 were included. The inclusion criteria were per relevant clinical practice guidelines [9, 10]. Regarding PBC, cases that met any two of the following three criteria were included: (1) elevated alkaline phosphatase (ALP) and gamma-glutamyl transpeptidase (GGT) levels, with extra- or intrahepatic bile duct obstruction excluded by imaging examination; (2) positivity for serum anti-mitochondrial antibodies and PBC-specific antinuclear antibodies as determined by immunofluorescence and/or specific anti-gp210/anti-sp100 testing; and (3) histological evidence of non-suppurative destructive cholangitis and small bile duct destruction. PBC cases were staged using the Ludwig system [11], whereas CHB was staged using the METAVIR scoring system [12]. PBC stages I and II and CHB stages F1 and F2 were defined as early stages, whereas PBC stages III and IV, and CHB stages F3 and F4 were defined as progressive stages.

### Ultrasonographic measurements

**Fig. 1:**
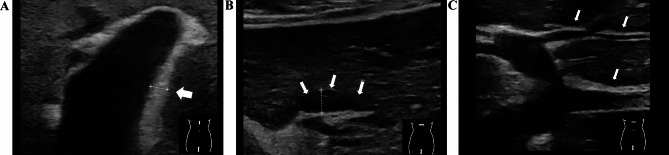
The measurement of PVW and PHB. (**A**) The measurement of PVW; (**B**-**C**) and the measurement of PHB is selected from the hypoechoic width around the ramus inferior segment of the left external lobe of portal vein

**Fig. 2:**
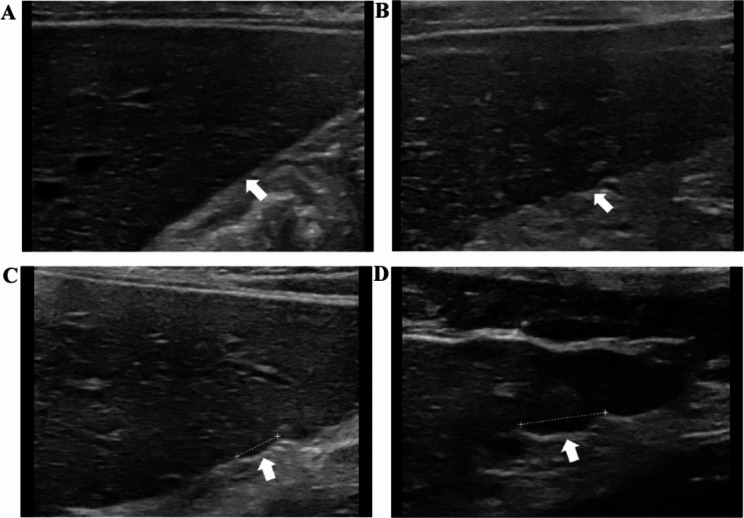
The ultrasound findings of the liver surface. (**A**) Smooth; (**B**) mild irregular (≤ 5 mm); (**C**) moderate irregular (6-10 mm); (**D**) severe irregular (> 10 mm)

**Fig. 3:**
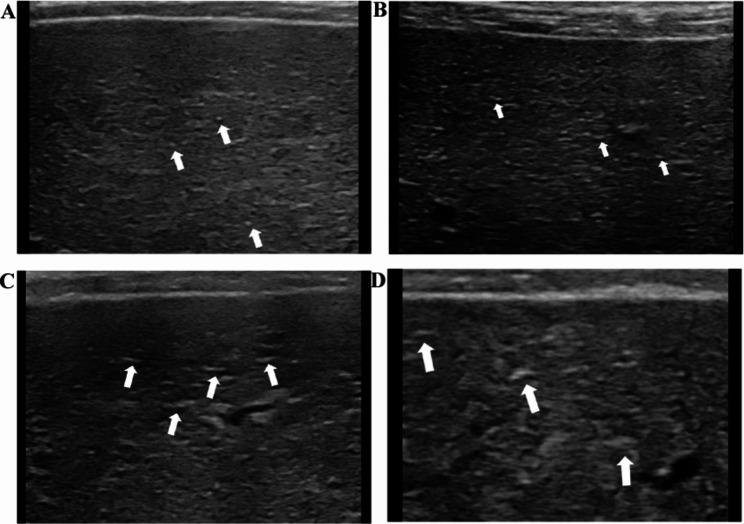
The ultrasound findings of the liver echo texture. (**A**) Fine (≤ 1 mm); (**B**) mildly coarse (2-5 mm); (**C**) coarse (> 5 mm); (**D**) small nodules (≤ 3 mm)

**Fig. 4:**
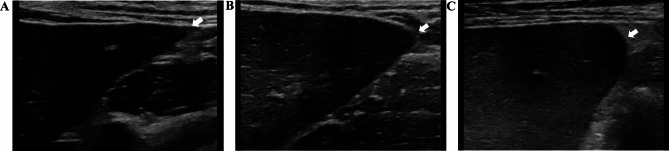
The ultrasound findings of the liver edge. (**A**) Sharp; (**B**) mildly dulled; (**C**) dulled

All the ultrasound images were obtained and analyzed by two sonographers with more than 10 years of experience in general sonography who were blind to the histological and clinical status of the patient. An AixPlorer general ultrasound machine (SuperSonic Imagine, France) with 1-6 MHz and 5-12 MHz probes was used to obtain the ultrasound images in this study. The following parameters were measured using the low-frequency ultrasound (LFUS) setting: the left hepatic lobe diameters (LHLD), portal and spleen veins; and the thickness and length of the spleen. In addition, the presence of gallbladder stones or polyps, abdominal lymph nodes, collateral circulation and ascites were recorded. LHLD was measured using the standard measurement section of the left liver lobe passing through the abdominal was defined as the maximum distance parallel to the midline of the body at the capsule of the top and bottom edges of the left liver lobe. The spleen area was estimated according to the following formula: 0.8 × spleen thickness × spleen length [13], by positioning the probe in the intercostal oblique position in the left upper flank. The spleen thickness was defined as the distance from the spleen hilum to the contralateral edge of the spleen. The spleen length was defined as the maximum distance from the top to the bottom pole of the spleen. Standard values were defined as follows: spleen area < 38 cm², LHLD ≤ 90 mm, portal vein diameter < 13 mm [14], and spleen vein diameter < 9 mm [15]. High-frequency ultrasound (HFUS) was used to evaluate the liver surface, echo texture, edge, and maximum thickness of the portal vein wall (PVW) by positioning the probe longitudinally below the xiphoid process. Figure 1a shows how the thickness of the longitudinal section of the sagittal part of the left portal vein was estimated. The width of the periportal hypoechoic band (PHB) was obtained by positioning the probe transversely below the xiphoid process. Figure 1b-c shows how the hypoechoic width around the ramus inferior segment of the left external lobe of the portal vein was obtained. Average values were obtained by repeating all the abovementioned measurements three times using magnified images. The liver surface and echogenicity were characterized not only by using reference criteria from previous studies [3, 16] but also by additional criteria that were defined during this study. The degree of undulation of the inferior surface of the right liver lobe between the hepatobiliary and hepatorenal sections was categorized as follows: (1) smooth; (2) mild irregular (≤ 5 mm); (3) moderate irregularity (6–10 mm); and (4) severe irregularity (> 10 mm; Fig. 2). Liver echo texture, determined by the average diameter of hyperechoic areas and nodules in the right lobe, was categorized as follows: (1) fine (≤ 1 mm); (2) mildly coarse (2–5 mm); (3) coarse (> 5 mm); (4) small nodules (≤ 3 mm) [17]; and (5) large nodules (> 3 mm; Fig. 3). The liver edge was categorized as follows [3]: (1) sharp ; (2) mildly dulled; and (3) dulled (Fig. 4). To assess inter-observer agreement between the two observers, parallel double-blind ultrasound examinations were performed in 10 patients with PBC (one at stage I, four at stage II, one at stage III, and four at stage IV), and inconsistencies in the assessment were discussed to reach a consensus.

### Statistical analysis

SPSS 22.0 statistical software was used for data analysis. Enumeration data were expressed as rates and analyzed using a chi-square test to compare the differences in each ultrasound image feature between disease stages. The measurement data were expressed as means ± standard deviations or medians. A t-test or rank-sum test was used for comparing the means of the two groups, and a one-way ANOVA or a non-parametric test was used for comparing the means between multiple groups. The degree of correlation of different parameters with the histological staging was determined using Spearman’s rank correlation analysis. A *p*-value < 0.05 was considered statistically significant.

The diagnostic performance of composite indices was determined using receiver operating characteristic (ROC) curves. Optimal cut-off values were chosen to maximize the sum of the sensitivity and specificity on the Youden index.

## Results

### Demographic and biochemical characteristics of the patients

A total of 75 and 57 patients with PBC and CHB respectively, were included in this study, with a median age of 50 (range 16–78) years. The PBC cohort included 12 patients at stage I, 25 at stage II, 13 at stage III, and 25 at stage IV, whereas the CHB cohort included 17 patients at stage F1, 13 at stage F2, 14 at stage F3, and 13 at stage F4. Fifty-one patients with PBC and 44 patients with CHB underwent a biopsy. Twenty-four stage IV patients with PBC and 13 stage F4 patients with CHB were clinically confirmed due to the risk of bleeding. Fifteen patients with PBC were excluded from the study due to the presence of comorbid liver disease from other causes, the unavailability of high-frequency measurements, or an insufficient portal area. The biochemistry results for all the included patients are summarized in Table 1. The agreement between the two observers in the characterization of the liver surface, edge, portal vein diameter, splenic vein diameter, LHLD, spleen area, PHB width, and PVW thickness was acceptable, with the intra-class correlation coefficients (ICCs) for each feature ranging from 0.739 (95% CI: 0.247–0.928) to 0.969 (95% CI: 0.880–0.992). However, the agreement between the two observers for liver echo texture was poor, with an ICC of 0.444 (95% CI: -0.215–0.826).

**Table 1:** The comparison of clinical and biological characteristics between PBC and CHB group

Variable	PBC (n = 75)	CHB (n = 57)	*P* value
Female, n (%)	68(90.7%)	36(63.2%)	< 0.001
Age, y	52(16–78)	47(25–61)	0.001
BMI	22(17–30)	22(18–30)	0.907
ALT, U/L	27.0(7.0-155.9)	26.0(8.0-405.0)	0.583
AST, U/L	46.0(13.4–210.0)	28.0(10.0-412.0)	< 0.001
TBIL, U/L	21.3(7.5–314.0)	15.9(3.0-72.5)	0.001
ALP, U/L	162.0(46.0-1110.0)	82.0(38.0-209.0)	< 0.001
GGT, U/L	89.0(5.0-1637.0)	20.0(6.0-715.0)	< 0.001
TBA, U/L	39.1(1.1-264.9)	4.8(0.6-114.4)	< 0.001

Data are shown as median (range). Abbreviation: BMI, body mass index; ALT, alanine aminotransferase; AST, aspartate aminotransferase; TBIL, total bilirubin; ALP, alkaline phosphatase; GGT, gamma-glutamyl transferase; TBA, total bile acid; PBC, primary biliary cholangitis; CHB; chronic hepatitis B

### Ultrasound imaging findings in PBC

**Fig. 5:**
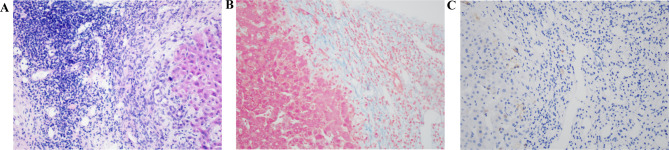
The histology of PHB in one patient after liver transplantation. (**A**) **H**/E stained shows many inflammatory cells in the PHB; (**B**) Masson stained shows enormous fibrosis in PHB; (**C**) CK7 immunohistochemistry shows mild cholestasis in the PHB

A PHB was observed in 93.3% of patients with PBC, and the median values of PHB in stages I to IV were 1.2 mm (range 0–1.8), 1.7 mm (range 1.0–2.7), 2.7 mm (range 1.0–3.9), and 4.2 mm (range 2.5–10.0), respectively. PHB width and PVW thickness were increased in progressive stages (*p* < 0.001). Ultrasound-guided puncture of the PHB in the left lobe of the transplanted liver was performed in one patient with stage IV PBC, and the biopsy revealed the presence of inflammatory cells, fibrous tissue, and mild cholestasis surrounding the portal triads (Fig. 5).

The LHLD was significantly increased in PBC stages II and III, with the percentage of increase from stages I to IV being 0% (0/12), 64.0% (16/25), 69.3% (9/13) and 16.0% (4/25), respectively. The mean values of the LHLD were 76.7 ± 8.7 mm, 91.4 ± 9.5 mm, 98.7 ± 18.5 mm, and 77.4 ± 24.4 mm for stages I to IV, respectively. The spleen area increased significantly in stages III and IV, with the median values being 45.7 cm^2^ (range 24.7–115.8) and 65.7 cm^2^ (range 29.8–138.5), respectively.

### Correlation between ultrasound imaging findings and histological staging in PBC

Histological staging was positively correlated with PHB width (r = 0.857, *p* < 0.001), PVW thickness (r = 0.488, *p* < 0.001), spleen area (r = 0.774, *p* < 0.001), spleen vein diameter (r = 0.713, *p* < 0.001), and portal vein diameter (r = 0.286, *p* = < 0.001). The diagnostic efficacy of applying all ultrasound image findings as the composite indices for progressive PBC (≥ stage 2) was very satisfactory, with an area under the ROC curve of 0.965, and sensitivity and specificity of 0.936 and 0.917, respectively. The diagnostic efficacy of the composite indices for advanced PBC (≥ stage 3) was also very good, with an area under the ROC curve of 0.926, and sensitivity and specificity of 0.807 and 0.917, respectively.

### Differences in ultrasound imaging findings between patients with PBC and CHB

**Fig. 6:**
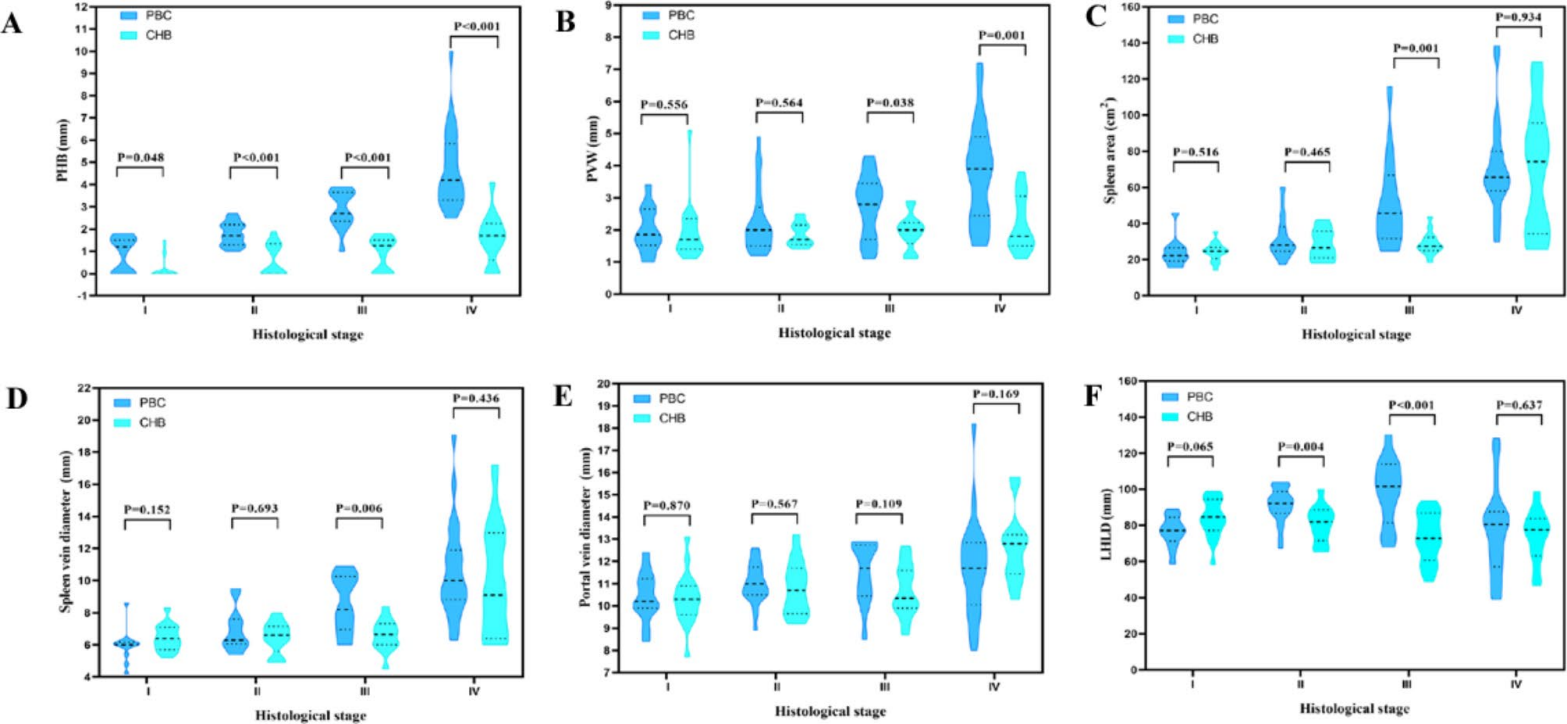
Violin plot showing the difference in ultrasound image between PBC and CHB patients. (**A**) PHB, (**B**) PVW, (**C**) spleen area, (**D**) spleen vein diameter, (**E**) portal vein diameter, (**F**) LHLD

As shown in Table 2, the PHB showed a higher rate and width in the PBC group compared to the CHB group, and the PVW was thicker in the PBC group compared to the CHB group for progressive stages. The splenic area and splenic vein diameter were higher in the PBC group at stage III than in the CHB group at stage F3, with increases of 69.2% and 46.2% in the PBC group and 14.3% and 0% in the CHB group, respectively. No difference was noted in the portal vein diameter between the two groups matched according to stage, and the LHLD from stage F2–F3 patients with CHB did not exhibit the typical enlargement observed in patients with PBC. (Fig. 6).

**Table 2:** Comparison of measurement values between PBC and CHB group

Group	LHLD	PVW	PHB	Spleen area	Portal vein diameter	Spleen vein diameter
PBC (stage I)	76.7 ± 8.7	1.9(1.0-3.4)	1.2(0-1.8)	22.3(15.5–45.8)	10.4 ± 1.1	6.0(4.2–8.6)
CHB (F0-1)	84.0 ± 10.9	1.7(1.1–5.1)	0(0-1.5)	24.6(14.3–35.6)	10.3 ± 1.2	6.4(5.2–8.3)
***P***	0.065	0.556	0.048	0.516	0.87	0.152
PBC (stage II)	91.4 ± 9.5	2.0(1.2–4.9)	1.7(1.0-2.7)	28.1(17.3–60.1)	11.0 ± 0.9	6.3(5.4–9.5)
CHB (F2)	80.7 ± 10.3	1.7(1.4–2.5)	0(0-1.9)	26.5(18.0-42.2)	10.8 ± 1.2	6.6(4.9-8.0)
***P***	0.004	0.564	< 0.001	0.465	0.567	0.693
PBC (stage III)	98.7 ± 18.5	2.8(1.1–4.3)	2.7(1.0-3.9)	45.7(24.7-115.8)	11.5 ± 1.4	8.2(6.0-10.9)
CHB (F3)	73.4 ± 13.8	2.0(1.1–2.9)	1.3(0-1.8)	27.3(18.4–43.7)	10.7 ± 1.2	6.7(4.5–8.4)
***P***	< 0.001	0.038	< 0.001	0.001	0.109	0.006
PBC (stage IV)	77.4 ± 24.4	3.9(1.5–7.2)	4.2(2.5–10.0)	65.7(29.8-138.5)	11.7 ± 2.1	10.0(6.3–19.1)
CHB (F4)	73.9 ± 14.8	1.8(1.1–3.8)	1.7(0-4.1)	74.3(25.6-129.5)	12.6 ± 1.6	9.1(6.0-17.2)
***P***	0.637	0.001	< 0.001	0.934	0.169	0.436

Data are shown as mean ± SD (range) or median (range). Abbreviation: PBC, primary biliary cholangitis; CHB, chronic hepatitis B; LHLD, Left hepatic lobe diameter; PVW, portal vein wall; PHB, periportal hypoechoic band

In the progressive stages, liver surface characteristics and echo texture differed between the two groups. While 44.0% of stage IV patients with PBC exhibited moderate irregularities in the liver surface, 69.2% of the stage F4 patients with CHB presented severe irregularities. The proportion of regenerative nodules was significantly lower in the PBC group at stage IV compared to those of the CHB group (5.3% vs. 44.4%). A complete comparison of ultrasound imaging findings between patients with PBC and CHB, discriminated by disease stage, is provided in Tables 3 and 4, as well as Supplementary Tables 1 and 2.

**Table 3:** The characteristics of liver ultrasound image in different histological stage with PBC patient

Characteristics	PBC (stage I) n = 12	PBC (stage II) n = 25	PBC (stage III) n = 13	PBC (stage IV) n = 25
**Liver surface**				
Smooth	7(58.3%)	6(24.0%)	3(23.1%)	1(4.0%)
Mild irregular (≤ 5 mm)	4(33.3%)	17(68.0%)	6(46.2%)	6(24.0%)
Moderate irregular (6-10 mm)	1(8.3%)	2(8.0%)	3(23.1%)	11(44.0%)
Severe irregular (> 10 mm)	0(0%)	0(0%)	1(7.7%)	7(28.0%)
**Liver echo texture**				
Fine (≤ 1 mm)	8(66.7%)	5(20%)	1(7.7%)	0(0%)
Mildly coarse (2-5 mm)	4(33.3%)	18(72%)	8(61.5%)	9(36%)
Coarse (> 5 mm)	0(0%)	1(4%)	3(23.1%%)	15(60%)
Small nodules (≤ 3 mm)	0(0%)	1(4%)	1(7.7%)	1(4.0%)
Large nodules (> 3 mm)	0(0%)	0(0%)	0(0%)	0(0%)
**Liver edge**				
Sharp	9(75.0%)	15(60%)	7(53.8%)	2(8%)
Mildly dulled	3(25.0%)	9(36%)	5(38.5%)	9(36%)
Dulled	0(0%)	1(4%)	1(7.7%)	14(56%)

Abbreviation: PBC, primary biliary cholangitis

**Table 4:** The characteristics of liver ultrasound image in different fibrosis stage with CHB patient

Characteristics	CHB (F1) n = 17	CHB (F2) n = 13	CHB (F3) n = 14	CHB (F4) n = 13
**Liver surface**				
Smooth	8(47.1%)	4(30.8%)	0(%)	0(0%)
Mild irregular (≤ 5 mm)	9(52.9%)	7(53.8%)	5(35.7%)	1(7.7%)
Moderate irregular (6-10 mm)	0(0%)	2(15.4%)	8(57.1%)	3(23.1%)
Severe irregular (> 10 mm)	0(0%)	0(%)	1(7.1%)	9(69.2%)
**Liver echo texture**				
Fine (≤ 1 mm)	6(35.3%)	2(15.4%)	0(0%)	0(0%)
Mildly coarse (2-5 mm)	11(64.7%)	8(61.5%)	2(14.3%)	1(7.7%)
Coarse (> 5 mm)	0(0%)	2(15.4%)	4(28.6%)	8(61.5%)
Small nodules (≤ 3 mm)	0(0%)	1(7.7%)	5(35.7%)	0(0%)
Large nodules (> 3 mm)	0(0%)	0(0%)	3(21.4%)	4(30.8%)
**Liver edge**				
Sharp	12(70.6.%)	9(69.2%)	4(28.6%)	0(0%)
Mildly dulled	5(29.4%)	3(23.1%)	4(28.6%)	3(23.1%)
Dulled	0(0%)	0(0%)	6(42.9%)	10(76.9%)

Abbreviation: CHB, chronic hepatitis B

## Discussion

Our investigation of ultrasound imaging findings in patients diagnosed with PBC went beyond the conventional markers characterized in previous studies. The scope of observation was expanded by taking advantage of both high- and low-frequency ultrasound measurements, which allowed us to uncover unique ultrasound findings in patients with PBC. Using this approach, we identified characteristic patterns of change in the LHLD that occur during PBC progression. Notably, we observed that the PVW thickness and PHB width were increased in more advanced PBC stages. These novel findings will be immensely valuable to enhance the assessment of disease progression in patients with PBC.

We observed the presence of PHB in the liver ultrasound images of patients diagnosed with either PBC or CHB. While the presence of a PHB alone is not a definitive indication of PBC, it is nevertheless more frequently present and wider in PBC cases, particularly as the disease progresses. During our investigation, we performed pathological analysis of a rare patient with PBC who underwent transplantation. Our findings revealed inflammatory cell infiltration, the presence of fibrous tissue, and mild cholestasis within the PHB range surrounding the portal triads. In the early stages of PBC, inflammation was minimal, and the degree of fibrosis was insignificant. However, as the disease progressed, the gradual damage to the bile duct epithelium led to the accumulation of lymphocytes in the portal triads, resulting in cholestasis, necrosis, and varying degrees of fibrosis. Therefore, we believe that it is more meaningful in the diagnosis of progressive stages of PBC according to the degree of widening of PHB. We speculated that the wider PHB observed in patients with PBC could be attributed to differences in the degree of inflammation and cholestasis around the portal triads. Furthermore, patients in our study having middle to advanced stages of PBC exhibited a thickened intrahepatic duct wall with enhanced echogenicity. The enhanced thickness of the intrahepatic duct wall was quantitatively confirmed in the study. Of note, the contrast between the PHB and thickened PVW may be confusing among observers and lead to misinterpretation of the enhanced echogenicity of the intrahepatic duct wall. Although the determination of enhanced echogenicity of the intrahepatic portal vein wall was subjective, this was additionally confirmed using MRI data. Moreover, a previous study by Kovač et al. reported that, of 44 patients with PBC, 72.7% exhibited periportal hyperintensity [6], which is consistent with our findings.

As PBC progresses, the excessive accumulation and toxic effect of bile acid can lead to a decline in liver cell function, resulting in the compensatory regeneration of the liver. In the early stages of the disease, approximately 70–80% of patients with PBC experience hepatomegaly, and in the end stage, approximately 20% develop splenomegaly [18]. A previous study reported that 75% of patients with PBC had hepatic left lobe enlargement, and splenomegaly was only observed in the presence of significant hepatic fibrosis [19], which is consistent with our findings. In our investigation, we not only identified hepatosplenomegaly in patients with PBC but also observed distinct patterns of changes in the LHLD and spleen area at different stages of the disease. Specifically, we observed a significant increase in LHLD at PBC stages II and III, and a significant increase in spleen area at stages III and IV. As PBC progresses, the portal vein diameter does not differ significantly from that observed at all stages of CHB; however, we noted that the increase in splenic area and the widening of the splenic vein occur earlier in PBC compared to patients with CHB. This suggests that portal hypertension may manifest earlier in these patients. Recent studies have demonstrated that portal hypertension in PBC can exhibit up to a 40% increase over 10-years [20], and it can occur earlier in the course of the disease, well before the rise in serum bilirubin and the development of cirrhosis. This phenomenon is linked to portal area and hepatic sinusoid lesions [21].

It has been reported that the liver surface, echogenicity, and liver edge of patients with chronic liver disease undergo regular changes as the disease progresses [3]. In our study, we noted similar changes in patients with PBC. Nonetheless, it is noteworthy that the liver surface of patients with PBC remained smoother than that of patients with CHB as the disease progressed. Furthermore, during the cirrhosis period, the ultrasound images of patients with PBC differed from those of most patients with chronic liver disease, who typically manifest more regenerative nodules in the liver parenchyma [22]. In our study, we observed that among the 25 patients with stage IV PBC, only 4% showed few regenerative nodules. In contrast, 30.8% of patients with stage F4 CHB presented nodular manifestations in their imaging data. In general, the progression of CHB to cirrhosis is characterized by macronodular cirrhosis, whereas regular micronodular cirrhosis dominates in PBC [18, 23]. Importantly, Aishima et al. proposed that the lower degree of interlobular bile duct loss in PBC could lead to macronodular cirrhosis with more acini, whereas severe interlobular bile duct loss could result in the separation of individual acini, leading to micronodular cirrhosis [23]. In this study, the incidence of intrahepatic nodules assessed by ultrasound imaging was lower in patients with PBC than in those with CHB. Two plausible reasons could account for this finding. First, 96% of patients with PBC with cirrhosis were identified based on clinical symptoms. Thus, their condition may have been severe at the time of diagnosis, and they might have already developed a substantial number of severe interlobular bile duct defects that could manifest as small nodular cirrhosis. Second, the size of the nodules in patients with PBC was either equal to or smaller than the liver lobules, implying that the ultrasound imaging resolution may not be sufficient to detect them. Patients with advanced disease often present with typical manifestations of portal hypertension, ascites, and collateral circulation. Our study findings showed that 20.0% of patients with PBC presented with gallbladder stones or polyps, while 12.0% had enlarged abdominal lymph nodes. Furthermore, approximately 60.9% of patients with advanced (stage IV) PBC developed esophagogastric varices (EGV), which is a higher percentage than what has been previously reported by Gao et al. (50.9%) [24]. This may be because our study only considered the incidence of EGV in patients with stage IV PBC.

The main methods for non-invasive PBC staging based on ultrasound elasticity are transient elastography, shear-wave elastography (SWE), and real-time elastography (RTE). The clinical guidelines from the European Association for the Study of the Liver (EASL) state that vibration-controlled transient elastography (VCTE) plays an irreplaceable role in determining disease progression in PBC. An international multi-center study of 3,985 patients with PBC showed that liver stiffness measurements were independently associated with poor clinical outcomes and that 8 kPa and 15 kPa were the optimal diagnostic thresholds for risk stratification into low-, intermediate-, and high-risk populations [25]. SWE reliably distinguishes between mild, moderate, and severe liver fibrosis and cirrhosis, with areas under the receiver operating characteristic curve of 95.3%, 87.4%, 85.3%, and 95.3%, respectively [26]. Koizumi et al. found that the diagnostic accuracy of RTE in the diagnosis of severe liver fibrosis was higher than that of VCTE [27]. The ultrasound image findings of PBC were correlated with histologic staging, and there was subjectivity in the observation of liver surface, echo texture, and edge; quantitative methods have been used to observe the characteristics of liver echo texture in the study, and the inter-observer concordance ICC was 0.739. It is believed that with the improvement of the resolution of ultrasound instrumentation as well as the observers’ observation of the liver through high-frequency ultrasound with a large sample size, it will definitely further improve the inter-observer Consistency. The consistency of observation for liver edge was poor, with an ICC of 0.444. In future studies, we will improve the observation method by applying quantitative measurement of liver edge angle instead of human competent observation. For this study, we preferred the measurement of PHB, PVW, LHLD and splenic area as observational indexes to determine the disease progression of PBC. The different elastography techniques have their advantages in the monitoring of PBC progression, and ultrasound measurement of two-dimensional images may provide a simple and quick complementary assessment method for a subset of patients with PBC whose elastography results may have been affected by factors such as rib space stenosis, elevated bilirubin, abdominal distension or abdominal wall thickness [5].

Our study had some limitations that need to be considered. Firstly, although we applied strict inclusion criteria, our sample size was relatively small, and the study was conducted in its early stages. Therefore, we need to expand our research by increasing the total number of participants and including additional healthcare centers, to explore the changes more comprehensively in ultrasound imaging according to the disease stage and identify additional distinctive ultrasound findings that can assist in this evaluation. Furthermore, we need to expand our research to determine whether these typical ultrasound findings are generalizable to other etiologies that cause liver progression. Secondly, our observers have received standardized training. For experienced ultrasonographers, the methods of measuring and observing ultrasound images in our study are relatively routine, but for less experienced ultrasonographers, they need to observe a certain number of cases and receive standardized training before they can be applied. Finally, elastography technique is currently a common non-invasive diagnostic method for predicting the progression of chronic liver disease in clinical practice. Our research did not involve the application of elastography technique in the diagnosis of PBC. In future research, we will expand the scope of our research, increase elastic measurement parameters, and further analyze the diagnostic value of ultrasound image features combined with elastography technique in the progression of PBC.

In summary, we conducted a thorough analysis of ultrasound imaging changes among patients with progressive PBC. Our findings revealed that this technique is a highly precise method for assessing the extent of disease progression in patients with PBC. Furthermore, our study suggests that ultrasound imaging may provide crucial clinical reference values for the prediction of the histological stage of the disease.

### Electronic supplementary material

Below is the link to the electronic supplementary material.

**Supplementary Material 1: Table 1**. The comparison of ultrasound image findings with PBC patient between different histological stages. **Table 2**. The comparison of ultrasound image findings with CHB patient between different fibrosis stages

## Data Availability

The datasets used and analyzed during the current study are available from the corresponding author on request.

## Abbreviations

AIH: Autoimmune hepatitis
ALP: Alkaline phosphatase
CHB: Chronic hepatitis B
EASL: European Association for the Study of the Liver
EGV: Esophagogastric varices
GGT: Gamma-glutamyl transferase
HFUS: High frequency ultrasound
ICC: Intra-class correlation coefficients
LFUS: Low frequency ultrasound
LHLD: The left hepatic lobe diameters
MRI: Magnetic resonance imaging
PBC: Primary biliary cholangitis
PHB: Periportal hypoechoic band
PSC: Primary sclerosing cholangitis
PVW: Portal vein wall
ROC: Receiver operating characteristic
RTE: Real-time elastography
SWE: Shear-wave elastography
VCTE: Vibration-controlled transient elastography
