# Supplementary material for: Ultrasound imaging findings in primary biliary cholangitis
Source: BMC Gastroenterol. 2023 Dec 19;23:448. doi: 10.1186/s12876-023-03083-w (PMC10729522; doi:10.1186/s12876-023-03083-w)
Supplement: Supplementary file 1 — Supplementary Material 1: Table 1. The comparison of ultrasound image findings with PBC patient between different histological stages. Table 2. The comparison of ultrasound image findings with CHB patient between different fibrosis stages [file 12876_2023_3083_MOESM1_ESM.docx]

**Supplementary**

**Table 1. The comparison of ultrasound image findings with PBC patient between different histological stages.**

| **Characteristics** | **PBC (stage I)** | **PBC (stage II)** | **PBC (stage III)** | **PBC (stage IV)** | ***P* value** |
| --- | --- | --- | --- | --- | --- |
|  | n=12 | n=25 | n=13 | n=25 |  |
| Liver surface | 1.0 (1.0-2.0) | 2.0 (1.5-2.0) | 2.0 (1.5-3.0) | 3.0 (2.0-4.0) ^ab^ | <0.001 |
| Liver echo texture | 1.0 (1.0-2.0) | 2.0 (2.0-2.0) | 2.0 (2.0-3.0) ^a^ | 3.0 (2.0-3.0) ^ab^ | <0.001 |
| Liver edge | 1.0 (1.0-1.8) | 1.0 (1.0-2.0) | 1.0 (1.0-2.0) | 3.0 (2.0-3.0) ^abc^ | <0.001 |
| LHLD | 76.7±8.7 | 91.4±9.5^a^ | 98.7±18.5^a^ | 77.4±24.4^c^ | 0.001 |
| PVW | 1.9 (1.0-3.4) | 2.0 (1.2-4.9) | 2.8 (1.1-4.3) | 3.9 (1.5-7.2) ^ab^ | <0.001 |
| PHB | 1.2 (0-1.8) | 1.7 (1.0-2.7) | 2.7 (1.0-3.9) ^a^ | 4.2 (2.5-10.0) ^ab^ | <0.001 |
| Spleen area | 22.3 (15.5-45.8) | 28.1 (17.3-60.1) | 45.7 (24.7-115.8) ^a^ | 65.7 (29.8-138.5) ^ab^ | <0.001 |
| Portal vein diameter | 10.4±1.1 | 11.0±0.9 | 11.5±1.4 | 11.7±2.1 | 0.092 |
| Spleen vein diameter | 6.0 (4.2-8.6) | 6.3 (5.4-9.5) | 8.2 (6.0-10.9) ^a^ | 10.0 (6.3-19.1) ^ab^ | <0.001 |

Note: The comparison of liver surface, liver echo texture and liver edge were given integral value according to observation standard. Abbreviation: PBC, primary biliary cholangitis; LHLD, Left hepatic lobe diameter; PVW, portal vein wall; PHB, periportal hypoechoic band. ^a^ P < .05 versus stage I; ^b^ P < .05 versus stage II; ^c^ P < .05 versus stage III.

**Table 2. The comparison of ultrasound image findings with CHB patient between different fibrosis stages.**

| **Characteristics** | **CHB (F1)** | **CHB (F2)** | **CHB (F3)** | **CHB (F4)** | ***P* value** |
| --- | --- | --- | --- | --- | --- |
|  | n=17 | n=13 | n=14 | n=13 |  |
| Liver surface | 2.0 (1.0-2.0) | 2.0 (1.0-2.0) | 3.0 (2.0-3.0) ^a^ | 4.0 (3.0-4.0) ^ab^ | <0.001 |
| Liver echo texture | 2.0 (1.0-2.0) | 2.0 (2.0-2.5) | 4.0 (3.0-4.3) ^ab^ | 3.0 (3.0-5.0) ^ab^ | <0.001 |
| Liver edge | 1.0 (1.0-2.0) | 1.0 (1.0-1.8) | 2.0 (1.0-3.0) ^ab^ | 3.0 (2.5-3.0) ^ab^ | <0.001 |
| LHLD | 84.0±10.9 | 80.7±10.3 | 73.4±13.8 | 73.9±14.8 | 0.064 |
| PVW | 1.7 (1.1-5.1) | 1.7 (1.4-2.5) | 2.0 (1.1-2.9) | 1.8 (1.1-3.8) | 0.748 |
| PHB | 0 (0-1.5) | 0 (0-1.9) | 1.3 (0-1.8) | 1.7 (0-4.1) ^a^ | 0.002 |
| Spleen area | 24.6 (14.3-35.6) | 26.5 (18.0-42.2) | 27.3 (18.4-43.7) | 74.3 (25.6-129.5) ^abc^ | <0.001 |
| Portal vein diameter | 10.3±1.2 | 10.8±1.2 | 10.7±1.2 | 12.6±1.6 ^abc^ | <0.001 |

Note: The comparison of liver surface, liver echo texture and liver edge were given integral value according to observation standard. Abbreviation: CHB, chronic hepatitis B; LHLD, Left hepatic lobe diameter; PVW, portal vein wall; PHB, periportal hypoechoic band. ^a^ P < .05 versus F1; ^b^ P < .05 versus F2; ^c^ P < .05 versus F3.
